# Field-Based Characterization of Peste des Petits Ruminants in Sheep in Romania: Clinical, Pathological, and Diagnostic Perspectives

**DOI:** 10.3390/vetsci12070679

**Published:** 2025-07-18

**Authors:** Romică Iacobescu-Marițescu, Adriana Morar, Viorel Herman, Emil Tîrziu, János Dégi, Kálmán Imre

**Affiliations:** 1 Department of Animal Production and Veterinary Public Health, Faculty of Veterinary Medicine, University of Life Sciences “King Mihai I” from Timisoara, 300645 Timisoara, Romania; romica.iacobescu-maritescu@usvt.ro (R.I.-M.); adrianamorar@usvt.ro (A.M.); emiltirziu@usvt.ro (E.T.); 2 Department of Infectious Disease and Preventive Medicine, Faculty of Veterinary Medicine, University of Life Sciences “King Mihai I” from Timisoara, 300645 Timisoara, Romania; viorelherman@usvt.ro (V.H.); janosdegi@usvt.ro (J.D.)

**Keywords:** peste des petits ruminants, sheep, Romania, RT-PCR, seroprevalence, pathology

## Abstract

Peste des petits ruminants is a highly contagious transboundary viral disease that poses a serious threat to small ruminant populations worldwide. In 2024, seven outbreaks of PPR were recorded in sheep flocks from Timiș County, marking the second confirmed incursions of peste des petits ruminants virus (PPRV) in Romania. This study aimed to document the clinical presentation, pathological findings, and diagnostic confirmation with these field outbreaks. Comprehensive field investigations were carried out between July and September 2024, including clinical examinations, post mortem analysis, serological screening, and molecular detection using reverse transcription polymerase chain reaction (RT-PCR). A total of 13,203 sheep were evaluated, with an overall mortality rate of 12.77%. Characteristic clinical signs included mucopurulent nasal discharge, oral erosions, respiratory distress, and diarrhea. Gross lesions observed during necropsy included hemorrhagic bronchopneumonia, bile-stained liver, catarrhal enteritis, and mucosal hemorrhages. Serological testing revealed flock-level seroprevalence rates ranging from 46.7% to 80.0%, with higher rates observed in older animals. RT-PCR confirmed PPRV infection in all affected flocks. Our findings provide strong evidence of virulent PPRV circulation in an area where the virus had not been reported before. The results highlight an urgent need to strengthen surveillance systems, enhance diagnostic capacity, and foster cross-border collaboration. These field-based insights can contribute to both national and international efforts aimed at controlling and ultimately eradicating the disease.

## Simple Summary

Peste des petits ruminants (PPR) is a highly contagious viral disease affecting sheep and goats, often resulting in severe illness and high mortality. In 2024, seven outbreaks of PPR occurred in sheep flocks in Timiș County, Romania, marking the second confirmed occurrence of the disease in the country. During field investigations conducted between July and September 2024, a total of 13,203 sheep were examined and 1687 (12.77%) died due to the infection. Affected animals displayed typical clinical signs, including mucopurulent nasal discharge, respiratory distress, oral erosions, and diarrhea. Laboratory confirmation was achieved through serological testing and molecular detection using reverse transcription polymerase chain reaction (RT-PCR), both of which verified PPR virus infection in all affected flocks. These findings indicate the emergence of a virulent PPR strain in a region previously free of the disease, underscoring the urgent need for enhanced surveillance, rapid diagnostics, and coordinated control measures to prevent further spread within Romania and neighboring countries.

## 1. Introduction

Peste des petits ruminants (PPR), commonly known as the “plague of small ruminants”, is a highly contagious and economically important transboundary viral disease, that primarily affecting sheep and goats [1]. The causative agent, Peste des Petits Ruminants Virus (PPRV), belongs to the genus *Morbillivirus* within the family *Paramyxoviridae* [2]. Based on partial sequence analysis of the fusion gene, PPRV is classified into four genetically distinct lineages (I, II, III, and IV) [3,4,5,6].

First described by Gargadennec and Lallane in 1942 in Côte d’Ivoire, PPR is characterized by high morbidity and mortality rates, often resulting in significant economic losses in affected regions [7,8]. Since its initial identification, the disease has spread widely across Africa, the Middle East, and much of Asia, with recent reports emerging from previously unaffected regions of Europe. The World Organisation for Animal Health (WOAH, formerly OIE) designates PPR as a notifiable disease due to its serious impact on food security, rural livelihoods, and livestock-based economies [9].

To date, PPR has been confirmed in more than 70 countries, affecting an estimated 1.7 billion small ruminants worldwide [10]. Lineages I, II, and III are predominantly found in Africa, while lineage IV is widespread in Asia. However, recent reports indicate the incursion of Asian lineage IV into several African countries and its progression toward southern Europe, particularly through Turkey [1,11,12].

The clinical manifestations of PPR in sheep vary depending on the virulence of the strain, the host’s immune status, and the presence of concurrent infections [10]. Common symptoms include high fever, ocular and nasal discharge, oral erosions, pneumonia, and profuse diarrhea, often leading to dehydration and death in severe cases [11,13]. However, the clinical presentation may overlap with that of other infectious diseases, complicating diagnosis in field conditions. Therefore, laboratory confirmation through molecular or serological testing is essential for the accurate identification of PPRV [14,15,16,17].

The first official detection of PPR within the European Union occurred in 2018 in Bulgaria, near the Turkish border, prompting immediate control measures including mass culling [18]. This detection triggered the application of the European Union’s regulatory framework for animal diseases, notably Regulation (EU) 2016/429 (‘Animal Health Law’), which provides comprehensive rules for the prevention, control, and eradication of transmissible animal diseases within the EU [19]. Specifically, PPR is classified under Commission Delegated Regulation (EU) 2018/1882 as a category A + D + E disease, requiring immediate notification and stringent control measures [20]. The subsequent containment and eradication efforts are governed by Commission Delegated Regulation (EU) 2020/687, which outlines detailed protocols for managing outbreaks of category A diseases, underscoring the EU’s commitment to safeguarding animal health and public safety [21]. The first official detection of PPR in Romania occurred in 2018 in Tulcea County, near the border with Bulgaria, following an outbreak that affected local sheep populations and prompted immediate control measures, including culling and movement restrictions. The emergence of the disease in an EU member state prompted urgent epidemiological and clinical investigations, underscoring the need for effective surveillance and swift response mechanisms within the EU’s stringent transboundary disease regulatory framework.

Romania has a significant small ruminant population, particularly in rural and economically vulnerable areas where sheep farming represents a key livelihood. The appearance of PPR in this context poses a direct threat not only to animal health and trade but also to regional food security and socio-economic stability.

Globally, PPR has been targeted for eradication by 2030 through coordinated efforts led by the Food and Agriculture Organization (FAO) and the WOAH [9,22,23]. In this context, the emergence of outbreaks in previously disease-free regions, such as Romania, presents a critical challenge to the progress of this global initiative.

This study presents detailed clinical and pathological findings from natural outbreaks of PPR in sheep flocks in Clopodia village, Timiș County, Romania, during 2024. Previous outbreaks of PPR have been reported in Greece, marking a critical stage in the disease’s spread into southeastern Europe. These events highlight the transboundary nature of PPR and reinforce the urgent need for enhanced surveillance and coordinated control efforts, particularly in border regions, to prevent further incursions into the European Union [24,25,26,27,28]. By documenting disease progression, clinical presentation, and diagnostic confirmation, this research aims to enhance understanding of PPR dynamics in newly affected areas. The findings are intended to inform veterinary authorities and policymakers in strengthening national and regional strategies for PPR surveillance, control, and prevention.

## 2. Materials and Methods

### 2.1. Description of the Study Area

**Figure 1:**
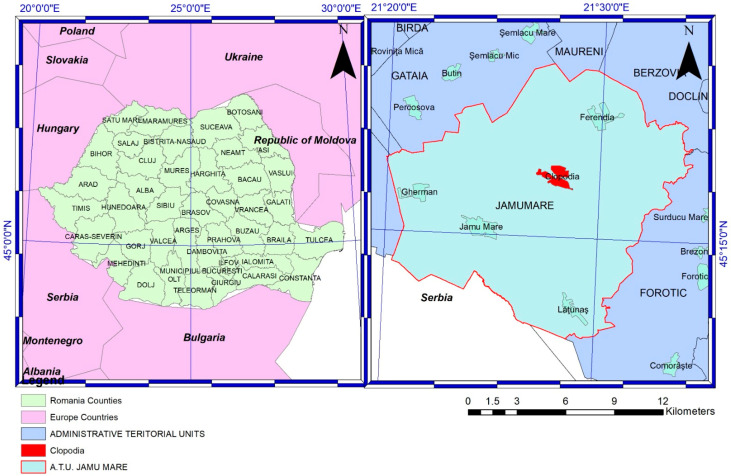
Geographical context of the 2024 PPR outbreaks in Romania. The map shows Romania (yellow background) in southeastern Europe, along with its neighboring countries (pink background). A detailed inset highlights the location of seven sheep flocks affected by peste des petits ruminants (PPR) outbreaks in Clopodia village (red background), part of the Jamu Mare commune (turquoise background), in relation to surrounding municipalities (blue background) within Timiș County.

The village of Clopodia (45.2831° N, 21.4665° E) (Figure 1) is located in the southeastern part of Timiș County, Romania, within the Bârzava Plain near the border with Caraș-Severin County, and forms part of the Jamu Mare commune. Situated in a small depression crossed by the Clopodia stream, a tributary of the Moravița River, the area features predominantly flat lowland terrain at an average altitude of approximately 150 m above sea level. The region has a temperate continental climate with sub-Mediterranean influences, characterized by warm summers and mild winters, favorable for livestock farming and agriculture. Soils are primarily chernozem, fertile and well-drained, supporting both crop cultivation and grazing. According to the 2022 census, Clopodia has a population of 788 inhabitants. The local economy is primarily based on agriculture and livestock farming, particularly sheep breeding. Timiș County holds the largest sheep population in Romania, with over 608,000 heads, approximately 24,591 of which are managed within Clopodia’s administrative territory. These flocks include both large-scale commercial operations and small-scale family farms.

### 2.2. Field Investigations

Between 26 July and 3 September 2024, seven outbreaks of a highly contagious disease affecting different sheep flocks were reported by herd owners across the study area. On the day of notification, veterinary authorities at both local and county levels conducted field visits to each affected flock to perform clinical assessments, collect epidemiological data, and obtain samples for laboratory investigations.

During each visit, flock owners were systematically interviewed to gather detailed information on the date of disease onset, total number of animals affected, primary clinical signs, treatment history, and distribution of cases by age and sex, and mortality data. Additionally, information on animal movements, recent introductions of new stock, and contact with neighboring flocks was recorded to assess potential sources and routes of transmission. Clinical examinations were performed on symptomatic animals to document the nature and severity of observed characteristic signs consistent with PPR. The affected flocks differed in terms of management systems, housing, and general herd constitution. The large-scale commercial flock (Flock 1) was managed under a semi-intensive system, with animals housed in enclosed facilities overnight and grazing during the day. In contrast, the small-scale flocks (Flocks 2–7) followed extensive systems, with continuous outdoor grazing and minimal confinement. Feeding practices also varied: the commercial operation supplemented grazing with concentrate feeds and stored forage, while smaller farms relied primarily on natural pasture with occasional hay supplementation, especially during dry periods. No systematic deworming schedules were reported in the majority of flocks, and subclinical parasitism (notably gastrointestinal nematodes) was suspected in at least three of the small-scale herds based on clinical interviews and veterinary records. No confirmed secondary bacterial infections or other co-infections were diagnosed during the investigation period, although access to laboratory testing for other pathogens was limited. These differences in environmental exposure, nutritional status, and baseline health may have contributed to the observed variability in mortality rates, with the highest case fatality occurring in the densely stocked commercial flock, likely due to rapid viral spread and higher animal contact rates

### 2.3. Specimen Collection for Diagnosis of PPR and Post Mortem Examination

For the PPR antibodies surveillance in clinically affected flocks, blood samples were collected from a statistically representative number of animals in each flock. Animals were selected to proportionally represent both symptomatic and asymptomatic individuals across different age groups, rather than through strict randomization, in accordance with field outbreak investigation protocols. Sampling design followed FAO and WOAH guidelines, aiming to detect seropositive individuals at a minimum expected prevalence of 10% with 95% confidence interval, as recommended for outbreak investigations in endemic and newly affected areas [24,25,26]. Accordingly, 30 animals were sampled in the large flock (>10,000), while proportional sampling, meaning 15 animals, was applied in smaller flocks (between 108 and 650 individuals), ensuring representative coverage across different age groups.

Nasopharyngeal and ocular swab samples were collected for confirmatory molecular laboratory diagnosis from two dead animals per flock, following the sampling procedures described in the Operational Manual for Intervention in Outbreaks of Pest of Small Ruminants provided by the National Veterinary Health and Food Safety Authority Accessed on 31 May 2025 (Available at: https://www.ansvsa.ro/blog/wpfb-file/manual-operational-pentru-interventia-in-focarele-de-pesta-rumegatoarelor-mici-pdf/). Tissue samples from the same animals included lung, mesenteric lymph nodes, spleen, and intestines for viral RNA detection.

All specimens were collected using sterile instruments, placed in appropriately labeled sterile containers, and stored under cool conditions (4–8 °C) during transport to the National Reference Laboratory for Animal Health and Diagnosis at the Institute of Animal Diagnostics and Health in Bucharest, Romania. Upon arrival, samples were processed on the same day or stored at −20 °C for further analysis.

Post mortem examinations were performed on recently deceased sheep suspected of PPR infection. Necropsies were conducted in accordance with standard veterinary biosafety procedures, and gross pathological changes were documented, with particular attention given to lesions consistent with PPRV [24].

### 2.4. Laboratory Investigations

#### 2.4.1. Detection of Antibodies Against PPRV in Serum Samples Using Serological (Competitive ELISA) Diagnostic Method

Serum samples were tested for antibodies against PPRV using the ID Screen^®^ PPR Competition ELISA kit (IDvet, Grabels, France), following the manufacturer’s instructions. This competitive ELISA utilizes monoclonal antibodies specific to the nucleoprotein (N) of PPRV and is recommended by the WOAH [26] for serological surveillance. Briefly, diluted serum samples and controls were added to microplate wells pre-coated with PPRV antigen and incubated. A horseradish peroxidase (HRP)-conjugated monoclonal antibody was then added, competing with antibodies in the sample for binding to the antigen. After washing steps, a substrate solution was added, and the enzymatic reaction was stopped after 15 min. Absorbance was measured at 450 nm. Results were interpreted based on the percentage of inhibition (PI) calculated by the kit software or manual instructions. Samples with PI values ≥ 50% were considered positive for anti-PPRV antibodies, indicating exposure or infection. Samples with PI values < 45% were considered negative, while those with values between 45% and 50% were regarded as doubtful and subjected to retesting.

#### 2.4.2. Molecular Reverse Transcription Polymerase Chain Reaction (RT-PCR) Technique

Total RNA was extracted from clinical samples using the QIAamp Viral RNA Mini Kit (Qiagen, Hilden, Germany) according to the manufacturer’s instructions. The extracted RNA was stored at −80 °C until further analysis. Detection of PPRV RNA was performed by reverse transcription polymerase chain reaction (RT-PCR) targeting the nucleoprotein (N) gene, as described by Couacy-Hymann et al. [29]. For complementary DNA (cDNA) synthesis, 1 µg of RNA was reverse-transcribed using M-MLV Reverse Transcriptase (Promega, Madison, WI, USA) in a 20 µL reaction volume, containing 1 µL of reverse primer NP4 (5′-CCTCCTCCTGGTCCTCCAGAATCT-3′), 4 µL of 5× reverse transcription buffer, 1 µL of 10 mM dNTP mix, 20 U of RNase inhibitor, and 200 U of M-MLV. The mixture was incubated at 42 °C for 60 min, followed by enzyme inactivation at 70 °C for 15 min. The PCR amplification was carried out using DreamTaq Green PCR Master Mix (2×) (Thermo Fisher Scientific, Waltham, MA, USA) in a 25 µL reaction containing 12.5 µL of master mix, 10 pmol each of forward primer NP3 (5′-TCTCGGAAATCGCCTCACAGACTG-3′) and reverse primer NP4, 2 µL of cDNA template, and nuclease-free water to final volume. Thermal cycling was performed in a Bio-Rad T100 Thermal Cycler under the following conditions: initial denaturation at 94 °C for 2 min; 35 cycles of 94 °C for 30 s, 55 °C for 30 s, and 72 °C for 1 min; followed by a final extension at 72 °C for 5 min. PCR products were resolved by electrophoresis on a 1.5% agarose gel stained with GelRed (Biotium, Fremont, CA, USA) and visualized under UV transillumination. The expected amplicon size was 351 base pairs, corresponding to a conserved region of the PPRV N gene. Each RT-PCR run included a known PPRV-positive RNA sample as positive control. Negative controls were included during both the RNA extraction phase (negative extraction control) and the amplification phase (no-template control) in each RT-PCR run to ensure diagnostic accuracy and prevent false-positive results. The RT-PCR assay used [29] is widely validated and recommended by WOAH for its high diagnostic sensitivity and specificity.

### 2.5. Statistical Analysis

Descriptive statistics were used to summarize flock size, mortality rates, and seroprevalence data. Mortality was expressed as the proportion of deceased animals relative to the total number of animals in each flock. Seroprevalence was computed as the proportion of animals testing positive for PPRV antibodies, stratified by age group (<24 months vs. ≥24 months).

To compare mortality rates across the seven flocks, the Kruskal–Wallis test was used due to the non-parametric data distribution and unequal sample sizes. Differences in seroprevalence between age groups were analyzed using Fisher’s exact test, suitable for categorical data with small group sizes. To evaluate the relationship between flock size and outbreak severity (as measured by mortality rate), Spearman’s rank correlation coefficient (ρ) was applied, considering the ordinal nature and non-normal distribution of the data. Statistical analyses were performed using GraphPad Prism version 9.0 (GraphPad Software, San Diego, CA, USA). A *p*-value < 0.05 was considered statistically significant.

## 3. Results

### 3.1. Clinical and Post Mortem Findings

Seven outbreaks of PPR occurred between late July and early September of 2024 in Clopodia village, Timiș County, Romania. A total of 13,203 sheep were present across the affected farms, with 1686 deaths, resulting in an overall mortality rate of 12.77%, although this rate varied widely between flocks (Table 1). The highest mortality rate (14.49%) was recorded in a large-scale commercial flock (flock 1), while the lowest (0.30%) occurred in a small-scale flock (flock 6).

**Figure 2:**
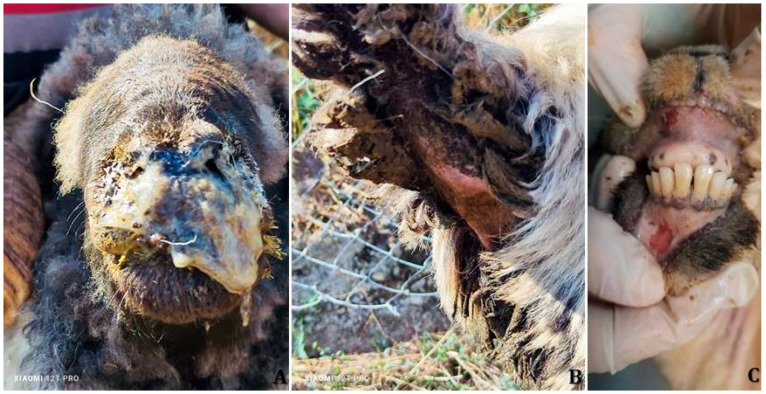
Representative clinical manifestations of PPRV infection in sheep. (**A**)—Mucopurulent nasal discharge, indicative of upper respiratory tract involvement. (**B**)—Fecal soiling of the perineal region, reflecting profuse diarrhea and gastrointestinal involvement. (**C**)—Red discoloration and localized lesions on the gingival mucosa, consistent with oral mucosal inflammation and hemorrhage characteristic of the mucosal phase of infection.

Clinical signs consistent with PPRV infection were observed in all seven affected sheep flocks. Affected animals commonly exhibited fever (>41 °C), depression, inappetence, excessive salivation, and erosive or ulcerative oral lesions. Respiratory signs were prominent and included dyspnea, coughing, respiratory distress, and mucopurulent nasal discharge (Figure 2A).

Ocular involvement manifested as conjunctivitis, epiphora, and mucous to purulent ocular discharge. Gastrointestinal signs included diarrhea (Figure 2B), and in more severely affected individuals, recumbency was noted. Ocular lesions and oral erosions with mucosal bleeding and pinpoint hemorrhages on the gingiva (Figure 2C) were also recorded by field veterinarians across multiple flocks.

**Figure 3:**
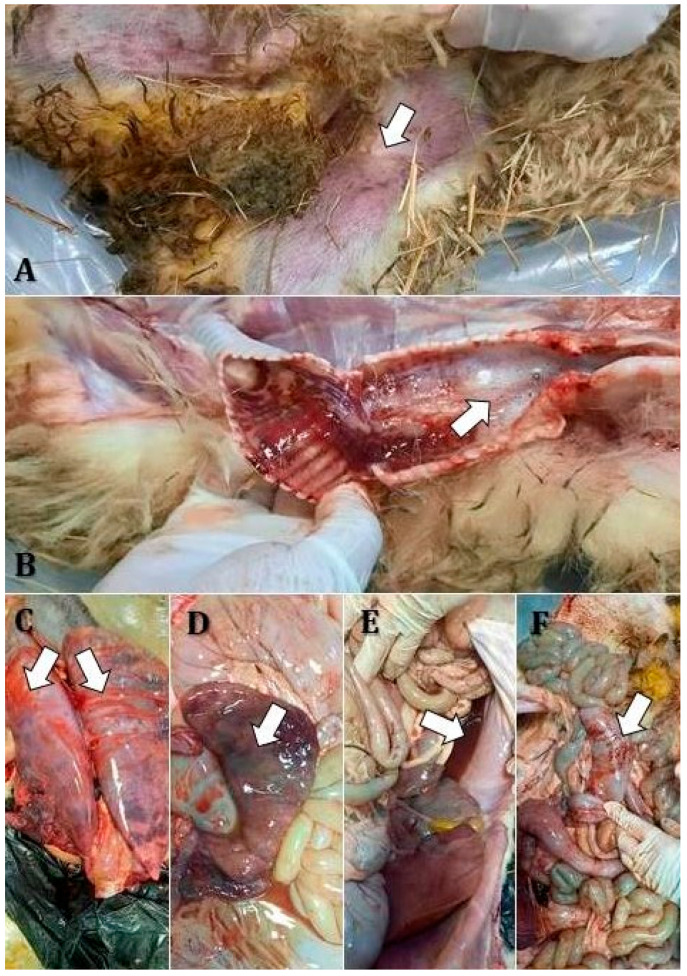
Gross pathological findings (indicated by white arrows) in a sheep naturally infected with PPRV: (**A**)—Cutaneous cyanosis in the inguinal region; (**B**)—Fibrinous exudate in the trachea; (**C**)—Hemorrhagic bronchopneumonia and fibrin deposits; (**D**)—Bile imbibition of the liver; (**E**)—Ascites and fluid accumulation in the forestomachs and intestines; (**F**)—Linear hemorrhages on the intestinal mucosa.

Post mortem examinations revealed several characteristic lesions, with varying severity among flocks. The corpses examined in flocks 1, 4, and 6 showed consistent findings including mucous nasal discharge, cutaneous cyanosis in the inguinal region (Figure 3A), frothy exudate in the trachea (Figure 3B), hemorrhages on the tracheal mucosa (Figure 3B), and hemorrhagic bronchopneumonia (Figure 3C).

Notably, there was pronounced lymphoid hyperplasia with increased reactivity in the submandibular and tracheobronchial lymph nodes. Digestive involvement included bile imbibition of the liver (Figure 3D), gallbladder ectasia, catarrhal enteritis, and liquid contents in the forestomachs and intestines (Figure 3E). The dead sheep in flock 2 presented gingival ulcerations, tracheal hemorrhages, and bronchopneumonia localized to the apical lobes. Edema and congestion were observed in the diaphragmatic lung lobes, along with ascites (Figure 3D), mesenteric lymph node hyperplasia, splenic congestion, and linear intestinal mucosal hemorrhages (Figure 3F).

Sheep from flocks 3 and 7 shared similar lesions, including lingual mucosal ulcerations, apical bronchopneumonia, diaphragmatic lobe congestion and edema, and ascites. There was reactive hyperplasia of the tracheobronchial and mesenteric lymph nodes, splenic congestion, and linear hemorrhages on the mucosal surface of the large intestine. The dead animals from flock 5 were characterized by petechial hemorrhages on the gingival mucosa, conjunctival mucosal congestion, and hemorrhagic bronchopneumonia affecting the apical lobes. Pulmonary edema and congestion of the diaphragmatic lobes were also noted. Hyperplastic lymphoreticulitis was evident in the tracheobronchial lymph nodes, accompanied by catarrhal enteritis, splenic congestion, and urinary retention.

### 3.2. Laboratory Results

Serological investigations based on the competitive ELISA technique conducted in all affected flocks revealed an overall seropositivity rate of approximately 61.9% (78/126). Seroprevalence varied by age group and flock. In animals aged under 24 months, the average seropositivity across flocks was approximately 47.5%, while in animals aged 24 months and older, the rate was markedly higher at 70.4%, suggesting increased prior exposure or longer time since infection in older animals.

Flock-level seropositivity ranged from 46.7% (Flock 5) to 80.0% (Flock 1), aligning with mortality trends in some cases, especially in the large-scale operation. These findings indicate variable infection dynamics among flocks and age groups, with larger farms potentially experiencing more severe outbreaks.

The individual-level serological data for results for each tested animal are available in the Supplementary Materials—Table S1.

All specimens submitted for laboratory molecular analysis, including both swab and tissue matrices, were tested for the presence of PPR viral RNA using reverse-transcription polymerase chain reaction (RT-PCR). The assay yielded positive results in all processed samples, confirming PPRV infection in each of the investigated flocks.

### 3.3. Statistical Comparisons

A statistically significant difference in mortality rates was found among the seven flocks (*p* < 0.01). Seroprevalence was significantly higher in animals aged ≥24 months compared to those <24 months (*p* = 0.03). A positive correlation (Spearman’s ρ = 0.79, *p* = 0.037) was observed between flock size and mortality rate, suggesting greater outbreak severity in larger farms.

## 4. Discussion

This study provides the first comprehensive clinical, pathological, serological, and molecular characterization of natural PPRV outbreaks in sheep in Clopodia village, Timiș County, Romania—an area previously considered free of the disease. The findings emphasize the pathogenic potential of PPRV in naïve ovine populations and highlight the epidemiological, diagnostic, and control challenges associated with its emergence in newly affected regions of Europe.

The clinical presentation observed in this study, including high fever, mucopurulent nasal and ocular discharge, oral erosions, and respiratory distress, aligns with classical descriptions of PPRV infection in sheep [4,12,13]. However, the severity and combination of signs, particularly the gastrointestinal involvement with diarrhea and oral hemorrhages, reinforce the hypothesis of infection with a virulent strain of lineage IV, which has been associated with more severe disease in both goats and sheep [1,10]. The variation in mortality rates among flocks was statistically significant (*p* < 0.01) and may reflect differences in the timing of disease detection, implementation of biosecurity measures, and the scale of operation. For example, the highest mortality was recorded in a large commercial flock, where delayed notification or higher animal density may have facilitated viral amplification. Furthermore, a positive correlation between flock size and mortality rate (Spearman’s ρ = 0.79, *p* = 0.037) indicates that large-scale operations may be at greater risk of severe outcomes, likely due to higher animal density, more frequent animal movements, and delayed containment measures. This underscores the need for prioritized surveillance, rapid response mechanisms, and tailored risk mitigation strategies in such high-density farm settings [1].

Notably, the high morbidity and case fatality rates recorded in the large-scale flock (14.49%) are consistent with reports from other previously PPR-free regions that recently experienced their first incursions of the virus [8,18].

Post mortem findings, such as hemorrhagic bronchopneumonia, bile imbibition of the liver, and linear intestinal hemorrhages, further support a systemic viral infection with a tropism for both the respiratory and digestive tracts, as reported in prior outbreaks involving virulent strains [3]. The presence of lymphoid hyperplasia in the tracheobronchial and mesenteric lymph nodes indicates a strong immune response, which may reflect either recent infection or partial immunological priming through natural exposure.

The overall seroprevalence of 61.9% recorded across the investigated flocks and using the competitive ELISA technique suggests widespread virus circulation within the affected area and indicates that a substantial proportion of animals had been exposed to PPRV by the time of sampling. This level of seropositivity is broadly consistent with findings from newly infected regions where the virus has spread rapidly within naïve populations prior to the implementation of control measures. The age-related disparity in seropositivity, with 70.4% in animals aged ≥24 months and 47.5% in those under 24 months, reflects a pattern commonly observed in endemic or recently infected areas. The significantly higher seroprevalence in animals aged ≥24 months compared to those <24 months (*p* = 0.03) suggests cumulative exposure among older animals or possibly an extended undetected circulation period of PPRV before the outbreaks were officially reported. Older animals are more likely to have survived previous exposure and mounted a detectable immune response, whereas younger animals, particularly those under one year, may still be in the period of susceptibility or may succumb to infection before seroconversion occurs [1]. While the sampling strategy was not fully randomized due to outbreak field constraints, proportional representation across age groups and clinical status was ensured to provide a practical estimate of within-flock exposure levels, as recommended by WOAH guidelines [26].

Comparable studies in other PPRV outbreaks have reported similar trends. For instance, Balamurugan et al. [17] documented a higher seroprevalence among older small ruminants in endemic regions of India, with values exceeding 75% in adult animals versus 40–50% in juveniles. Likewise, Kihu et al. [30] observed seropositivity rates of up to 68% in unvaccinated sheep in Kenya, with older animals showing significantly higher antibody levels due to cumulative exposure.

In contrast, in fully naïve populations experiencing a first wave of infection, lower seroprevalence (30–50%) is often recorded, particularly during early-stage outbreaks before peak transmission [8,12]. In this context, the relatively high seroprevalence observed in the Clopodia outbreaks, especially in flocks 1 and 6, may indicate that the virus had been circulating undetected for several weeks prior to official notification. This is further supported by the significant number of animals that tested positive despite showing no clinical signs at the time of sampling [31].

Importantly, flock-level variation from 46.7% to 80% suggests heterogeneity in transmission dynamics, which may relate to differences in management practices, flock density, biosecurity, or the timing of virus introduction. Higher seropositivity in large-scale operations may reflect both increased contact rates among animals and delayed intervention, resulting in wider spread before control measures were applied [31].

These findings emphasize the value of serological surveys not only for outbreak confirmation but also for reconstructing the temporal and spatial dynamics of infection within and between flocks. In the absence of vaccination (as in Romania’s context), seropositivity can be directly interpreted as evidence of natural infection, making it a crucial tool for epidemiological mapping and risk-based surveillance [32].

RT-PCR molecular detection of PPRV RNA in all sampled flocks confirms the viral etiology and validates the diagnostic approach recommended by WOAH for outbreak confirmation. Given the overlapping clinical features of PPR with other small ruminant diseases such as bluetongue or contagious pustular dermatitis (Orf disease), molecular confirmation remains essential for accurate diagnosis and disease reporting [16,33,34]. While clinical severity and regional epidemiology suggest the likely involvement of lineage IV PPRV, definitive lineage identification through genetic sequencing was not performed and represents a key objective for future outbreak investigations.

The emergence of PPR in Romania poses significant challenges for disease control within the European Union. The incursion of lineage IV into southeastern Europe likely reflects transboundary animal movement and underscores the need for harmonized regional surveillance systems. In this context, Romania’s geographic proximity to endemic areas and its substantial sheep population make it particularly vulnerable to future incursions or endemic establishment if containment efforts fail. Although the outbreaks described were detected and investigated rapidly, the study highlights the importance of farmer awareness, veterinary training, and diagnostic capacity in the early recognition and response. Moreover, the data support the urgent implementation of strategic vaccination, movement control, and active surveillance, especially in border regions and areas with high sheep density [35].

The detection of PPR in a previously unaffected area suggests likely introduction via cross-border movement of infected animals, possibly through informal trade routes or seasonal livestock movements. Though currently undocumented in Romania, wildlife reservoirs may also play a role and warrant further investigation. To mitigate future outbreaks, several key measures are recommended. Strategic vaccination in high-risk or border areas could be considered, particularly in line with FAO/WOAH guidance [23,26]. Enhanced farm-level biosecurity, including quarantine, controlled access, and hygiene protocols, is essential—especially in large-scale operations, which showed greater outbreak severity in this study.

In addition, movement control and animal traceability systems should be strengthened to support rapid response and epidemiological tracking. Finally, the implementation of active, risk-based surveillance—supported by regional coordination and data sharing—remains critical for early detection and containment. These integrated approaches will support both national resilience and the broader goal of global PPR eradication by 2030.

A limitation of the current study is the absence of whole-genome sequencing of the viral strains involved, which would enable more precise phylogenetic placement and tracking of the virus’s origin. Future investigations should also explore the role of wildlife reservoirs and potential co-infections that may affect disease severity.

## 5. Conclusions

The present study documents the first confirmed field outbreaks of PPRV in Romania, providing strong clinical, pathological, and molecular evidence of infection with a virulent lineage IV strain. The consistent clinical signs and post mortem findings, together with 100% RT-PCR positivity and widespread seroconversion, confirm active virus circulation and acute disease. Age-related seroprevalence patterns and inter-flock variability suggest complex transmission dynamics influenced by flock management and possible delays in detection. These findings highlight Romania’s vulnerability to PPRV incursion and the critical need for enhanced surveillance, rapid diagnosis, and coordinated control strategies. Importantly, the emergence of PPRV in southeastern Europe poses a serious threat to the global goal of eradication by 2030, underscoring the urgency of cross-border collaboration, veterinary capacity building, and targeted awareness efforts in high-risk regions.

## Figures and Tables

**Table 1:** Summary of PPR outbreaks in seven sheep flocks in Clopodia village, Timiș County, Romania, 2024: flock characteristics, mortality, and seropositivity rates by age group.

No. of Flock	Data of Notification	Type of Farm	No. of Animals	No. of Dead	Mortality Rate (%)	Seropositive Animals/Examined (%)		
						<24 Months	≥24 Months	Total
1.	26.07.2024	large scale	11,243	1641	14.49	8/12 (66.7)	16/18 (88.9)	24/30 (80.0)
2.	26.08.2024	small scale	623	26	4.17	4/6 (66.7)	6/9 (66.7)	10/15 (66.6)
3.	29.08.2024	small scale	228	7	3.07	3/4 (75.0)	6/11 (54.5)	9/15 (60.0)
4.	29.08.2024	small scale	108	2	1.85	2/7 (28.6)	7/8 (87.5)	9/15 (60.0)
5.	31.08.2024	small scale	501	5	0.99	3/9 (33.3)	4/6 (66.7)	7/15 (46.7)
6.	31.08.2024	small scale	329	2	0.30	4/5 (80.0)	7/10 (70.0)	11/15 (73.3)
7.	03.09.2024	small scale	171	3	1.75	2/5 (40.0)	6/10 (60.0)	8/15 (53.3)

## Data Availability

The data are contained within the article and the Supplementary Materials.

## Supplementary Materials

The following supporting information can be downloaded at: https://www.mdpi.com/article/10.3390/vetsci12070679/s1, Table S1: The individual-level serological data results.

## References

[1] Balamurugan V., Kumar K.V., Dheeraj R., Kurli R., Suresh K.P., Govindaraj G., Shome B.R., Roy P. (2021). Temporal and Spatial Epidemiological Analysis of Peste Des Petits Ruminants Outbreaks from the Past 25 Years in Sheep and Goats and Its Control in India. Viruses.

[2] ICTV Morbillivirus International Committee on Taxonomy of Viruses (ICTV). https://ictv.global/.

[3] Baron M.D., Diallo A., Lancelot R., Libeau G. (2016). Peste des Petits Ruminants Virus. Adv. Virus Res..

[4] Soltan M.A., Abd-Eldaim M.M. (2014). Emergence of peste des petits ruminants virus lineage IV in Ismailia Province, Egypt. Infect. Genet. Evol..

[5] Mantip S., Sigismeau A., Shamaki D., Woma T.Y., Kwiatek O., Libeau G., Farougou S., Bataille A. (2022). Molecular epidemiology of peste des petits ruminants virus in Nigeria: An update. Transbound. Emerg. Dis..

[6] Couacy-Hymann E., Berete K., Odoom T., Zerbo L.H., Mathurin K.Y., Kouakou V.K., Doumbouya M.I., Balde A., Ababio P.T., Ouoba L.B. (2023). The Spread of Peste Des Petits Ruminants Virus Lineage IV in West Africa. Animals.

[7] Gargadennec L., Lalanne A. (1942). La Peste Des Petits Ruminants. Bull. Serv. Zoo. Epizoot. AOF.

[8] Jones B.A., Rich K.M., Mariner J.C., Anderson J., Jeggo M., Thevasagayam S., Cai Y., Peters A.R., Roeder P. (2016). The economic impact of eradicating peste des petits ruminants: A benefit-cost analysis. PLoS ONE.

[9] OIE (2015). FAO Manual on Global Strategy for the Control and Eradication of Peste des Petits Ruminants. http://www.fao.org/3/a-i4460e.pdf.

[10] Dou Y., Liang Z., Prajapati M., Zhang R., Li Y., Zhang Z. (2020). Expanding Diversity of Susceptible Hosts in Peste Des Petits Ruminants Virus Infection and Its Potential Mechanism Beyond. Front. Vet. Sci..

[11] Kamel M., El-Sayed A. (2019). Toward peste des petits virus (PPRV) eradication: Diagnostic approaches, novel vaccines, and control strategies. Virus Res..

[12] Legnardi M., Raizman E., Beltran-Alcrudo D., Cinardi G., Robinson T., Falzon L.C., Djomgang H.K., Okori E., Parida S., Njeumi F. (2022). Peste des Petits Ruminants in Central and Eastern Asia/West Eurasia: Epidemiological Situation and Status of Control and Eradication Activities after the First Phase of the PPR Global Eradication Programme (2017–2021). Animals.

[13] Fathelrahman E.M., Reeves A., Mohamed M.S., Ali Y.M.E., El Awad A.I., Bensalah O.K., Abdalla A.A. (2021). Epidemiology and Cost of Peste des Petits Ruminants (PPR) Eradication in Small Ruminants in the United Arab Emirates-Disease Spread and Control Strategies Simulations. Animals.

[14] Shaila M.S., Purushothaman V., Bhavasar D., Venugopal K., Venkatesan R.A. (1989). Peste des petits ruminants of sheep in India. Vet. Rec..

[15] Singh R.K., Balamurugan V., Bhanuprakash V., Sen A., Saravanan P., Pal Yadav M. (2009). Possible control and eradication of peste des petits ruminants from India: Technical aspects. Vet. Ital..

[16] Singh R.P., Saravanan P., Sreenivasa B.P., Singh R.K., Bandyopadhyay S.K. (2009). Prevalence and distribution of peste des petits ruminants virus infection in small ruminants in India. Rev. Sci. Tech..

[17] Balamurugan V., Hemadri D., Gajendragad M.R., Singh R.K., Rahman H. (2014). Diagnosis and control of peste des petits ruminants: A comprehensive review. Virus Dis..

[18] Hacıoğlu S., King S., Çizmeci Ş.G., Yeşil Ö., Flannery J., Baron M.D., Batten C., Rajko-Nenow P.Z. (2020). Complete Genome Sequence of a Lineage IV Peste des Petits Ruminants Virus from Turkey, 2018. Microbiol. Resour. Announc..

[19] (2016). Regulation (EU) 2016/429 of the European Parliament and of the Council of 9 March 2016 on transmissible animal diseases and amending and repealing certain acts in the area of animal health (“Animal Health Law”). Off. J. Eur. Union.

[20] (2018). Commission Delegated Regulation (EU) 2018/1882 of 27 June 2018 supplementing Regulation (EU) 2016/429 of the European Parliament and of the Council as regards the list of diseases, categorization of diseases, and the list of animal species related to those diseases. Off. J. Eur. Union.

[21] (2020). Commission Delegated Regulation (EU) 2020/687 of 17 December 2019 supplementing Regulation (EU) 2016/429 of the European Parliament and of the Council as regards rules for the eradication and containment of certain diseases. Off. J. Eur. Union.

[22] Albina E., Kwiatek O., Minet C., Lancelot R., Servan de Almeida R., Libeau G. (2013). Peste des Petits Ruminants, the next eradicated animal disease?. Vet. Microbiol..

[23] Njeumi F., Ferrari G., Raizman E., Diallo A., Domenech J., Leboucq N., Munstermann S. (2015). Global Strategy for the Control and Eradication of PPR.

[24] PPR Laboratory Network (2025). Update on PPR Emergence in Europe. https://www.ppr-labs-oie-network.org/news/update-on-ppr-emergence-in-europe.

[25] WOAH (2024). Terrestrial Animal Health Code, Chapter 14.9: Infection with Peste des Petits Ruminants Virus.

[26] WOAH (2024). Manual of Diagnostic Tests and Vaccines for Terrestrial Animals.

[27] Cameron A.R., Baldock F.C. (1998). A new probability formula for surveys to substantiate freedom from disease. Prev. Vet. Med..

[28] Eloiflin R.-J., Grau-Roma L., Python S., Mehinagic K., Godel A., Libeau G., Summerfield A., Bataille A., García-Nicolás O. (2022). Comparative pathogenesis of peste des petits ruminants virus strains of difference virulence. Vet. Res..

[29] Couacy-Hymann E., Roger F., Hurard C., Guillou J.P., Libeau G., Diallo A. (2002). Rapid and sensitive detection of peste des petits ruminants virus by a polymerase chain reaction assay. J. Virol. Methods.

[30] Kihu S.M., Gachohi J.M., Ndungu E.K., Gitao G.C., Bebora L.C., John N.M., Wahome R.G. (2015). Seroepidemiology of peste des petits ruminants virus infection in Turkana County, Kenya. BMC Vet. Res..

[31] Balamurugan V., Varghese B., Muthuchelvan D., SowjanyaKumari S., Vinod Kumar K., Dheeraj R., Govindaraj G., Suresh K.P., Hemadri D., Roy P. (2020). Seroprevalence of Peste des petits ruminants in small ruminants in the North Eastern Region of India. Vet. Ital..

[32] Balamurugan V., Govindaraj G.N., Rahman H. (2016). Planning, implementation of peste des petits ruminants control programme and strategies adopted for disease control in India. Br. J. Virol..

[33] Balamurugan V., Saravanan P., Sen A., Rajak K.K., Venkatesan G., Krishnamoorthy P., Bhanuprakash V., Singh R.K. (2012). Prevalence of peste des petits ruminants among sheep and goats in India. J. Vet. Sci..

[34] Dundon W., Diallo A., Cattoli G. (2020). Peste des petits ruminants in Africa: A review of currently available molecular epidemiological data, 2020. Arch. Virol..

[35] Parida S., Muniraju M., Mahapatra M., Muthuchelvan D., Buczkowski H., Banyard A.C. (2015). Peste des petits ruminants. Vet. Microbiol..

